# A Multimodal Fatigue Detection System Using sEMG and IMU Signals with a Hybrid CNN-LSTM-Attention Model

**DOI:** 10.3390/s25113309

**Published:** 2025-05-24

**Authors:** Soree Hwang, Nayeon Kwon, Dongwon Lee, Jongman Kim, Sumin Yang, Inchan Youn, Hyuk-June Moon, Joon-Kyung Sung, Sungmin Han

**Affiliations:** 1 Bionics Research Center, Biomedical Research Division, Korea Institute of Science and Technology (KIST), Seoul 02792, Republic of Korea; srhwang@kist.re.kr (S.H.); 9kwon@kist.re.kr (N.K.); dw2@kist.re.kr (D.L.); jmkim0127@kist.re.kr (J.K.); s.yang@kist.re.kr (S.Y.); iyoun@kist.re.kr (I.Y.); crescent@kist.re.kr (H.-J.M.); 2 School of Biomedical Engineering, Korea University, Seoul 02841, Republic of Korea; 3 Division of Bio-Medical Science & Technology, KIST School, Korea University of Science and Technology, Seoul 02792, Republic of Korea; 4 KHU-KIST Department of Converging Science and Technology, Kyung Hee University, Seoul 02447, Republic of Korea

**Keywords:** physical fatigue detection, surface electromyography (sEMG), inertial measurement unit (IMU), hybrid deep learning, CNN-LSTM-attention, gait kinematics

## Abstract

Physical fatigue significantly impacts safety and performance across industrial, athletic, and medical domains, yet its detection remains challenging due to individual variability and limited generalizability of existing methods. This study introduces a multimodal fatigue detection system integrating surface electromyography (sEMG) and inertial measurement unit (IMU) signals, processed through a hybrid convolutional neural network–long short-term memory–attention (CNN-LSTM-Attention) model. Fatigue was induced in 35 healthy participants via step-up-and-down exercises, with gait data collected during natural walking before and after fatigue. The model leverages sEMG from the gastrocnemius lateralis and IMU-derived jerk signals from the tibialis anterior and rectus femoris to classify fatigue states. Evaluated using leave-one-subject-out cross-validation (LOSOCV), the system achieved an accuracy of 87.94% with bilateral EMG signals and a balanced recall of 87.94% for fatigued states using a combined IMU-EMG approach. These results highlight the system’s robustness for personalized fatigue monitoring, surpassing traditional subject-dependent methods by addressing inter-individual differences.

## 1. Introduction

Physical fatigue is a pervasive issue in modern society, stemming from muscle activity, energy depletion, and physical overexertion, profoundly impacting the musculoskeletal and metabolic systems and significantly impairing both physical and mental health [1]. Excessive workloads and prolonged physical activities, such as manual labor or intense exercise, can reduce attention, delay reaction times, and elevate the risk of accidents [2,3], while long-term effects may include chronic conditions such as cardiovascular disease, diabetes, and burnout [4]. Therefore, effectively detecting physical fatigue is essential for mitigating the immediate risk of accidents and preventing long-term health issues [5].

Traditional methods of fatigue assessment rely on subjective approaches such as self-report questionnaires, Likert scales, and Borg’s rating of perceived exertion (RPE), which lack reliability and quantifiability. Recent advancements in wearable technology have facilitated objective assessments using physiological signals such as EMG, electrocardiography (ECG), electrocardiography, and motion/gait data [6,7,8]. For instance, Lee et al. developed a wrist-worn ECG sensor paired with a critical power model to continuously estimate whole-body fatigue in construction workers, achieving high accuracy in field conditions by integrating personalized physiological thresholds with heart rate reserve signals [9]. Similarly, Antwi-Afari et al. proposed a machine learning framework that classifies physical fatigue into four levels using plantar pressure and acceleration data from a wearable insole system [10].

These studies represent notable progress in wearable-based fatigue monitoring. However, these studies often require specific postures or movements for fatigue analysis (e.g., rebar tying, squats, Pilates, treadmill, knee extensions), using data measured under those conditions, making it difficult to apply them to everyday life. Additionally, their intra-subject validation limits generalization to broader populations [10,11,12,13,14].

Traditional sensor fusion techniques, such as Kalman filters and particle filters, have been widely used to integrate multimodal data streams from sensors like IMU and EMG. These classical approaches effectively reduce noise and enhance signal reliability by leveraging prior knowledge about the smoothness and temporal continuity of physiological and kinematic signals [15,16]. For example, Kalman filters have been applied to fuse EMG and torque signals for muscle fatigue tracking [17]. Additionally, a study demonstrated that combining received signal strength indicator (RSSI) signals from body-worn sensors with IMU data using a Kalman filter improved body part tracking accuracy by approximately 50% compared to using RSSI signals alone [18].

However, while Kalman filters are computationally efficient and easy to implement, they have limitations when applied to nonlinear systems. In contrast, particle filters can handle nonlinear and non-Gaussian systems but are computationally expensive and suffer from issues like particle degeneracy and difficulty in determining the optimal number of particles [15]. Recently, AI-based approaches have gained traction as alternatives to traditional methods. These methods do not rely on predefined models or prior assumptions but instead learn patterns directly from raw data, offering sufficient flexibility to capture complex nonlinear relationships [15].

Kakhia et al. highlighted the potential of integrating multimodal physiological signals with deep learning to process complex datasets and deliver reliable, high-performance solutions. Research on deep learning for fatigue detection has explored various approaches [6]. For instance, studies have utilized sEMG sensors with a CNN-LSTM-Transformer hybrid model to classify muscle fatigue [19], demonstrating high accuracy but demanding substantial computational resources due to the multi-head attention layer and feed-forward layer. Similarly, Kinect sensors paired with RNN models have been applied to gait-based fatigue detection [20], showing promise but limited by spatially restrictive equipment. Other studies have integrated force and IMU sensors with SVM models to detect fatigue during physical tasks [21]. Additionally, machine learning techniques such as Random Forests, Gradient Boosting Machines, and LSTMs have been used to analyze EMG or IMU gait data for fatigue classification [22,23]. Despite their contributions, these studies are often constrained by controlled laboratory settings, such as treadmill experiments, reliance on subject-specific evaluations, or the use of restrictive hardware, which limits their broader applicability.

Furthermore, many prior studies have combined fatigue induction and detection within the same activity, producing task-specific outcomes with limited external applicability. These studies typically focus on specific environments or controlled settings, making it difficult to generalize the results to everyday activities. Additionally, the heavy reliance on subject-specific models has hindered the broad applicability of these approaches across diverse populations, as these models may not perform well for individuals outside of the original training set. Research on fatigue assessment based on natural gait and inter-subject validation is lacking. Addressing these gaps is essential for developing a fatigue detection framework applicable to everyday environments.

To address these challenges, this study proposes a novel approach by applying a CNN-LSTM-Attention hybrid model for fatigue detection during daily walking. Unlike previous methods, our approach prioritizes practical applicability by effectively detecting fatigue using EMG and IMU signals collected during over-ground walking, an activity that is simple and commonly performed in daily life, without relying on complex feature extraction steps. This study not only introduces a new system but also systematically validates it. To achieve this, our approach incorporates the following key features:

1. Natural Gait Evaluation: Fatigue was induced through a step-up-and-down task, and gait data were collected during natural over-ground walking. This approach captures valid fatigue-related gait changes that reflect real-world ambulatory conditions.
2. Multimodal Integration: A CNN-LSTM-Attention hybrid model fuses EMG and IMU data to analyze spatial–temporal signals and fatigue markers, enhancing accuracy and streamlining feature extraction and classification through deep learning.
3. Inter-subject Validation: LOSOCV accounts for individual variability [24], addressing the subject-dependent constraints of prior work.
4. Sensor Optimization: Systematic comparison of EMG and IMU configurations identifies an optimal setup for fatigue detection.

This paper is organized as follows: Section 2 describes the materials and methods used in this study, including participant procedure, data preprocessing, signal combinations, classification, and evaluation methods. Section 3 presents the results of the analysis. In Section 4, we discuss the findings and their implications. Finally, Section 5 concludes the study and suggests directions for future research.

## 2. Materials and Methods

### 2.1. Participant Procedure

Thirty-five healthy participants (17 males, 18 females; mean age: 32.14 ± 9.73 years, age range: 20–60) were recruited. All participants self-reported being free from neurological, musculoskeletal, or cardiovascular disorders. Written informed consent was obtained from all participants prior to the study, which was approved by the Institutional Review Board of the Korea Institute of Science and Technology (IRB No. KIST-202209-HR-013).

Since the boundary between fatigued and non-fatigued states can be ambiguous, in the experiment setup, we defined the state without performing any task as the pre-fatigue state, and the state after performing a physical fatigue-inducing task with an RPE score of 17 or higher, indicating “very hard” exertion [25], as the post-fatigue state. Fatigue was induced by having participants perform repeated step-up-and-down tasks using a 20 cm step box, with participants adjusting a metronome to their self-selected walking pace to maintain a consistent speed. This process continued until their RPE score reached 17 or higher. Fatigue levels were quantified using self-reported RPE scores before and after exercise.

**Figure 1:**
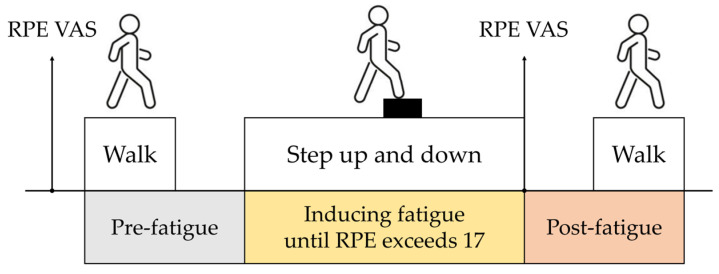
Experimental procedure schematic. Stages include (1) pre-fatigue gait collection (25 m corridor), (2) fatigue induction (20 cm step box, RPE ≥ 17), and (3) post-fatigue gait collection (25 m corridor).

Data collection occurred in two stages: (1) pre-fatigue stage, where participants walked back and forth along a 25 m straight corridor at a self-selected pace to establish baseline gait data; and (2) post-fatigue stage, where gait data were collected again immediately after fatigue induction along the same 25 m corridor (Figure 1).

To simultaneously measure gait kinematics and muscle activity, wireless EMG and IMU sensors (Trigno Avanti, Delsys Inc., Natick, MA, USA), which are widely adopted in previous studies for measuring muscle activation and gait kinematics [26,27], were used.

Sampling rate mismatches between IMUs can lead to angular calculation errors of up to 200% of the actual value and timestamp losses, resulting in distorted time intervals between samples [28,29]. In gait analysis, such mismatches cause temporal asynchrony across multiple IMUs, leading to misdetection of gait events and cumulative errors that degrade the accuracy of temporal and spatial gait parameters.

To mitigate these issues, we synchronized the sensors using version 3.6.0 of the Trigno Control Utility (Delsys Inc., Natick, MA, USA), a standalone software platform that seamlessly manages the Trigno Wireless Biofeedback System. This software pairs up to 16 Trigno Avanti sensors with the Base Station, ensuring inter-sensor delays of less than one sample period via a proprietary RF protocol [26].

**Figure 2:**
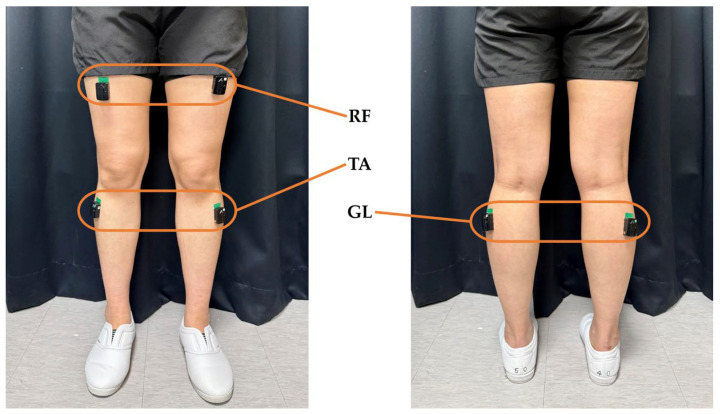
Placement of EMG and IMUs. Six sensors were positioned bilaterally on the tibialis anterior, lateral gastrocnemius, and rectus femoris.

Sensors were attached to the tibialis anterior (TA), lateral gastrocnemius (GL), and rectus femoris (RF) muscles of both lower limbs (Figure 2). These sensors integrate EMG (sampling rate: 1259 Hz) and IMUs (sampling rate: 148 Hz), enabling the capture of electromyographic signals from the lower limb muscles and the motion of the lower limbs during gait. The experiment was conducted in a controlled laboratory environment to ensure consistent sensor placement and walking conditions.

### 2.2. Data Preprocessing

Raw sensor data from the IMU and EMG were preprocessed to ensure high-quality input for the classification model. Outliers exceeding ±3 standard deviations were removed, and missing data due to sensor dropout were filled using linear interpolation. Gyroscope signals were band-pass filtered between 0.25 and 30 Hz to isolate gait-related frequencies, while accelerometer data were filtered between 1 and 20 Hz [30]. EMG signals were band-pass filtered between 20 and 500 Hz to preserve muscle signal integrity [31].

To enhance gait characteristic analysis, IMU data were processed to compute Linear Jerk (the rate of change of acceleration) and Angular Jerk (the rate of change of angular acceleration) (Equations (1) and (2)) [32]. These metrics captured abrupt movements and rotational changes, supporting the assessment of motion smoothness and stability.

**Figure 3:**
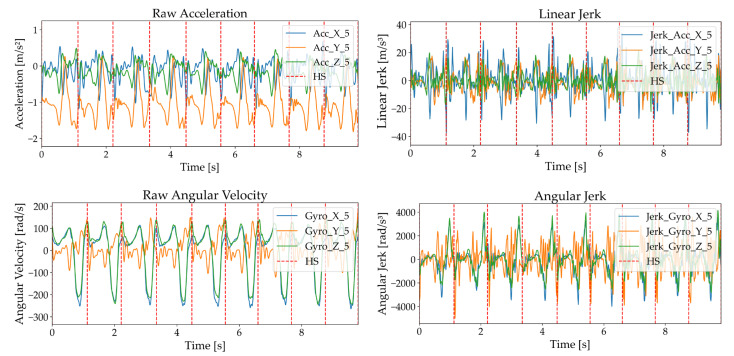
Acceleration and angular velocity signals with heel strike markers, including raw acceleration, Linear Jerk, raw angular velocity, and Angular Jerk.

**Figure 4:**
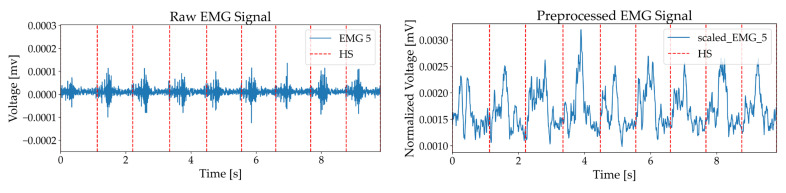
EMG with heel strike markers, including raw EMG and rectified signal.

For the IMU signal, a 100-millisecond moving average filter was applied to reduce noise and highlight motion trends, while for the rectified EMG signal, it was used to eliminate steep amplitude spikes and clarify muscle activation patterns, producing smoothed signals suitable for analysis [33,34]. Figure 3 and Figure 4 illustrate these processes, displaying acceleration, angular velocity, and EMG signals with heel strike markers alongside their processed outputs. Finally, all data were resampled to 148 Hz to enable integrated analysis of IMU and EMG data.

(1) $$Linear\;Jerk=\frac{d}{dt}a\left(t\right)=\frac{a\left(t+dt\right)-a(t)}{dt}$$

(2) $$Angular\;Jerk=\frac{d}{dt}\omega \left(t\right)=\frac{\omega \left(t+dt\right)-\omega (t)}{dt}$$

Preprocessed signals were segmented into 3 s windows with 50% overlap and were normalized with z-score to ensure consistency across participants and signal types. Finally, the gait dataset collected from 35 participants in non-fatigued and fatigued states was composed of 35 participants × 2 fatigue types × 9 windows. Each segmented dataset has the form of 630 total segments × 444 frames × 6 sensors, serving as standardized input data for analysis and learning.

### 2.3. Signal Combinations

In this study, the lower limbs were equipped with six sensors ($TA_{L},\;TA_{R},\;GL_{L},\;GL_{R},RF_{L},\;RF_{R})$ to collect biomechanical data, including 3-axis Linear Jerk, 3-axis Angular Jerk, and EMG signals. These sensors enabled the extraction of spatiotemporal features for fatigue state classification, with performance evaluated across various sensor signal combinations.

The signal combinations were designed based on the functional roles of specific muscles and gait kinematics. The GL sensor measuring the gastrocnemius muscle was predominantly affected by muscular exertion during step-up-and-down tasks and provided time-series data on fatigue-induced changes in muscle activation through EMG signals. The TA sensor, located on the tibialis anterior (shank), and the RF sensor, attached to the rectus femoris (thigh), utilized both accelerometer and gyroscope signals to capture time-series data on the linear and rotational movements of the shank during gait. This study integrated EMG data from the GL sensor with IMU data from the TA and RF sensors to collect signals related to muscle activity and gait kinematics, utilizing these signals for fatigue state classification to optimize classification accuracy.

As detailed in Table 1, the signal combinations were systematically categorized based on sensor locations and signal types. Combinations 1 and 2 assess individual sensor features derived from a single low limb, whereas Combination 3 evaluates a bilateral configuration. Combinations 4 through 6 incorporate IMU-derived features collected from identical sites on both $TA_{L}$ and $TA_{R}$. Combinations 7 through 9 utilize features from sensors attached to $RF_{L}$ and $RF_{R}$. Combinations 10 through 13 analyze features from sensors attached to different sites on a single leg, while Combination 14 includes features from all attached sensors. This approach allowed for a comprehensive evaluation of model performance under various sensor configurations, utilizing multimodal data to enhance fatigue state classification accuracy.

### 2.4. Classification

In this study, a CNN-LSTM-Attention hybrid model was developed to classify fatigue states during everyday ambulation, utilizing multimodal sEMG and IMU signals, including accelerometer and gyroscope measurements. CNNs can automatically extract spatial features from raw sensor data, such as muscle contraction intensities from sEMG and gait parameters from IMU, eliminating the need for manual feature engineering [35]. LSTMs model temporal dependencies, learning changes in sequential data, such as time-varying patterns in signals or sequences, over extended periods [35]. The self-attention mechanism enhances the model’s precision by dynamically prioritizing critical time-series segments [36], such as transitions from non-fatigued to fatigued states, where subtle shifts in muscle activation or gait patterns are most pronounced.

By integrating spatial, temporal, and contextual information from sEMG and IMU data, this hybrid approach could offer improved robustness and generalizability compared to standalone deep learning models (e.g., CNN or LSTM) or traditional machine learning methods (e.g., SVM). Unlike computationally intensive Transformer-based models [19], which demand substantial data and processing power, Attention-based models are well suited for resource-constrained wearable devices due to their relatively low computational complexity and memory usage.

The CNN-LSTM-Attention model was selected for gait-based fatigue assessment based on several advantages. It supports the processing of high-dimensional multimodal data and is well suited to capture subject-specific and temporally evolving variations in muscle activation and kinematic patterns. The integration of feature extraction and classification within the model also minimizes the need for manual signal segmentation or handcrafted feature selection, thereby simplifying the analytical pipeline.

The CNN component consists of two convolutional layers with 64 and 128 filters, respectively, to extract spatial features from the time-series data of EMG, accelerometer, and gyroscope signals. Renowned for their ability to automatically learn hierarchical patterns, CNNs are well suited for processing multivariate sensor data. These layers, which are combined with max-pooling, identify key spatial characteristics of gait and muscle activity essential for distinguishing non-fatigued from fatigued states.

Next, the LSTM network, with 64 units, captures temporal dependencies within the sequential data. Its strength lies in modeling long-term patterns that evolve with fatigue progression, enabling effective differentiation of fatigue states over time.

**Figure 5:**
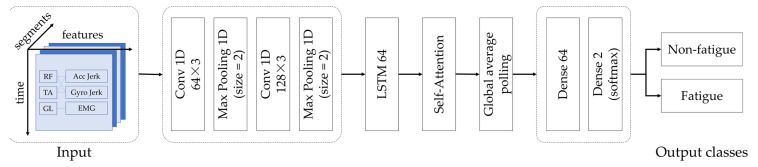
CNN-LSTM-Attention framework for multimodal fatigue detection.

A self-attention mechanism further enhances the model by dynamically weighting the most relevant segments of the time-series data. Unlike fixed-window approaches, this layer focuses on critical moments, such as fatigue onset or recovery, improving the detection of subtle changes in gait and muscle signals. The output is then globally pooled and passed through dense layers, culminating in a binary classification output via a softmax activation. These components are detailed in Figure 5.

The hybrid model was trained on participants’ EMG and IMU signals, to classify non-fatigued versus fatigued states in a binary setting. By integrating these complementary data sources, the model potentially enhances its generalization and predictive capability across diverse fatigue scenarios. Comprehensive validation of this approach is detailed in the evaluation section. All analyses were performed using Python version 3.12.7

### 2.5. Evaluation

The hybrid CNN-LSTM-Attention model was rigorously evaluated using LOSOCV for subject-independent performance. In this approach, data from one subject were reserved for testing, while the model was trained on data from all remaining subjects, iterating across all participants. This method ensured the model’s ability to generalize to unseen individuals, a key requirement for practical fatigue monitoring systems. The training process incorporated an 80:20 split of the dataset into training and validation sets. Hyperparameters, including learning rate, number of layers, and attention weights, were optimized using early stopping and learning rate reduction to minimize validation loss.

Subject-dependent performance was assessed using a 9-fold cross-validation strategy, where each participant’s data were divided into nine folds based on balanced group assignments within non-fatigued and fatigued states. Eight folds were used for training and one for testing, repeated across all folds, providing a robust measure of the model’s consistency within individual subjects.

The LOSOCV evaluation was supported by a detailed analysis of confusion matrices and classification metrics. Performance was quantified using accuracy, precision, recall, and F1-score (Equations (3)–(6)), with average test values calculated across folds to assess overall effectiveness and reliability in distinguishing non-fatigued from fatigued states.

(3) $$Accuracy=\frac{TP+TN}{TP+FP+TN+FN}$$

(4) $$Precision=\frac{TP}{TP+FP}$$

(5) $$Recall=\frac{TP}{TP+FN}$$

(6) $$F1\;score=2\times \;\frac{Precision\times Recall}{Precision+Recall}$$

## 3. Results

The hybrid CNN-LSTM-Attention model classified fatigue states using multimodal EMG and IMU signals. Table 2 presents the classification performance of the model across 14 distinct combinations of sensor and signal types, reporting both subject-independent and subject-dependent accuracy metrics. In subject-independent conditions, an analysis of the initial three configurations revealed that bilateral EMG signals from the gastrocnemius lateralis outperformed unilateral signals, highlighting the value of bilateral muscle activity (Comb. 1–3). Configurations utilizing Linear Jerk and Angular Jerk components from the tibialis anterior showed that combined jerk signals performed better than single-modality signals (Comb. 4–6). Among configurations using Linear Jerk and Angular Jerk from the rectus femoris, the one relying solely on Linear Jerk demonstrated superior performance (Comb. 7–9). In configurations combining EMG from the gastrocnemius lateralis with motion data, the setup integrating EMG with tibialis anterior jerk signals outperformed those with rectus femoris components (Comb. 10–12). However, a configuration that further incorporated both tibialis anterior and rectus femoris jerk signals alongside EMG achieved even higher accuracy than the previous standout (Comb. 13).

When including all combinations that exhibited superior performance within each bilateral signal category (Comb. 14), the results were somewhat lower than those with bilateral EMG signals (Comb. 3) and the single leg hybrid (Comb. 10). Across all combinations, subject-dependent accuracy consistently exceeded subject-independent accuracy, indicating that user-specific reference data significantly enhances performance. Furthermore, subject-dependent accuracy tended to increase with greater feature complexity. In contrast, subject-independent accuracy did not scale similarly, as higher accuracy was often achieved with fewer features. For instance, while Comb. 14 recorded 0.8444 with 20 features, Comb. 3 achieved 0.8794 with 2 features, Comb. 10 reached 0.8587 with 7 features, and Comb. 13 attained 0.8698 with 10 features, all outperforming Comb. 14. This suggests that optimizing fatigue detection accuracy in subject-independent scenarios requires effective signal combinations that integrate jerk and EMG signals complementarily, rather than simply increasing feature count.

**Figure 6:**
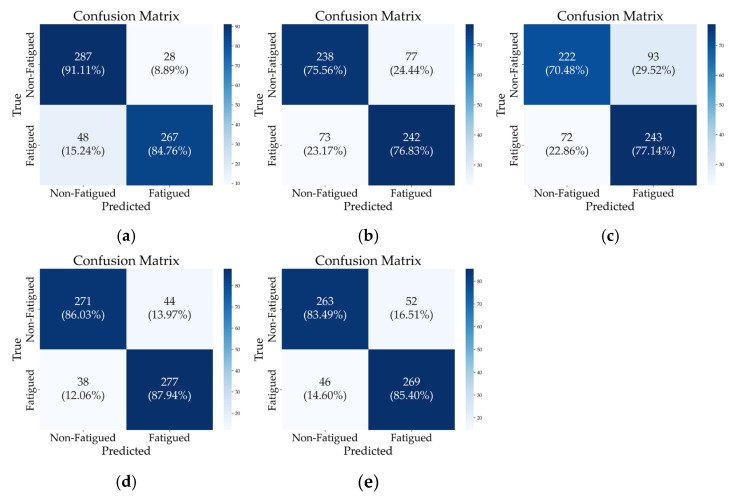
Confusion matrices for selected signal combinations under subject-independent (LOSOCV) conditions. (**a**) Comb. 3, (**b**) Comb. 6, (**c**) Comb. 7, (**d**) Comb. 13, (**e**) Comb. 14.

Confusion matrices and performance metrics (Figure 6 and Table 3) facilitate a detailed analysis of classification performance, demonstrating the ability of each configuration to distinguish between non-fatigued and fatigued states across various sensor setups. Accordingly, five configurations, each exhibiting superior performance within their respective categories, were selected for focused comparison. Bilateral GL EMG signals achieved the highest accuracy of 0.8794 with minimal features (two) in the EMG-only category (Comb. 3). Next, the single-leg hybrid representative, integrating IMU and EMG, achieved an accuracy of 0.8698 (Comb. 13), followed by the configurations that exhibited superior performance within each bilateral signal category, with an accuracy of 0.8444 (Comb. 14). In contrast, TA-based and RF-based jerk setups performed lower than EMG-inclusive configurations (Comb. 6 and 7).

Comparing the top two configurations, Comb. 13 exhibits a lower recall for non-fatigued states than Comb. 3 (0.8603 vs. 0.9111) but surpasses it in fatigue detection recall (0.8794 vs. 0.8476). This indicates that Comb. 13 reduces false negatives for fatigue cases, which is particularly important in fatigue monitoring systems where failing to detect fatigue could lead to safety risks or reduced performance. In contrast, while Comb. 3 shows higher accuracy for the non-fatigued class, it comes at the cost of a higher false negative rate for fatigued individuals.

Additionally, although Comb. 14 utilizes a large feature set, it underperformed compared to other configurations, suggesting that feature complexity alone does not guarantee enhanced class-specific performance.

The bottom two configurations emphasize the limitations of an IMU-only approach (Comb. 6 and 7). This analysis underscores the importance of balancing sensor modality and feature count, providing key insights into selecting an optimal sensor configuration for accurate and reliable fatigue detection.

In summary, based on Table 3, Comb. 13 achieves not only high accuracy (0.8698) but also a critical advantage in minimizing false negatives for fatigue (recall of 0.8794 for the fatigued class), establishing it as an optimal combination. Moreover, while using EMG alone can make gait detection challenging in everyday situations, combining it with IMU signals enhances its utility [37,38,39].

**Figure 7:**
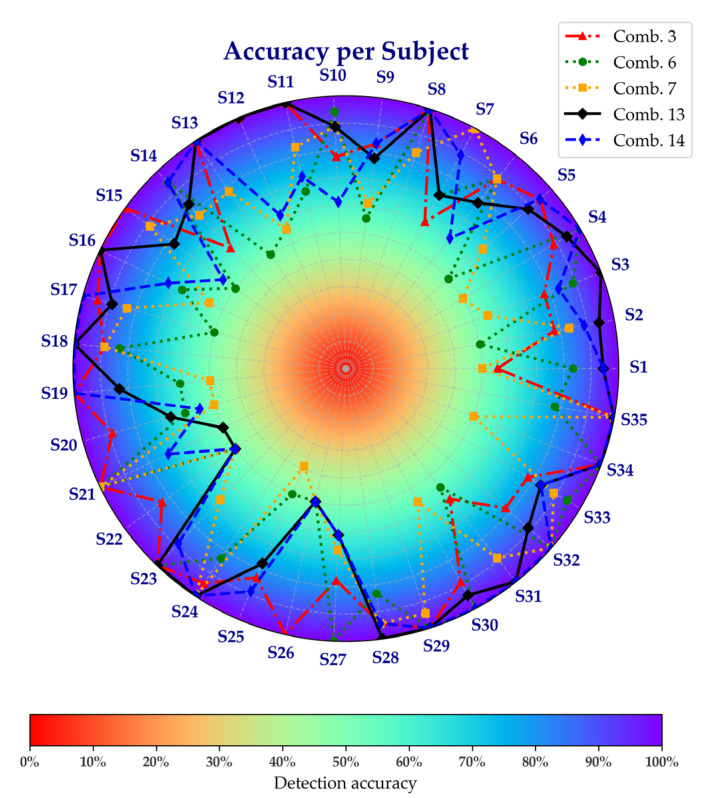
Accuracy per subject across selected sensor combinations.

To further explore the performance of these combinations at an individual level, Figure 7 presents a radial chart of per-subject accuracy. This chart shows that the accuracy of the subjects varies depending on the combination of signals chosen, with some combinations improving performance and others decreasing it. These results demonstrate that the contribution of IMU and EMG signals to fatigue detection varies across individuals.

**Figure 8:**
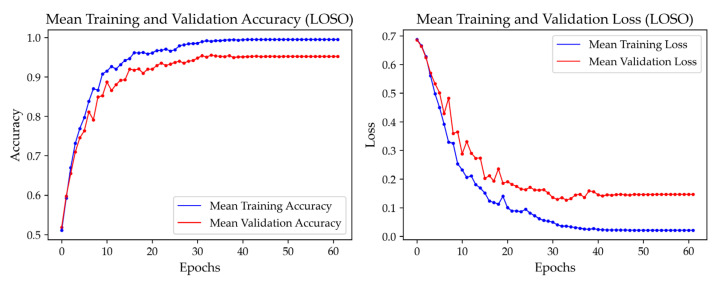
Training and validation accuracy and loss curves for the hybrid CNN-LSTM-Attention model using Comb. 13.

Figure 8 evaluates the model’s stability with Comb. 13, presenting the training and validation accuracy and loss curves for the hybrid CNN-LSTM-Attention model. These curves demonstrate stable convergence, with training accuracy reaching 0.9950 and validation accuracy stabilizing at 0.9517, reflecting robust learning without overfitting. The corresponding loss curves consistently decline, converging at 0.0212 for training and 0.1466 for validation, confirming the effectiveness of optimized hyperparameters.

**Figure 9:**
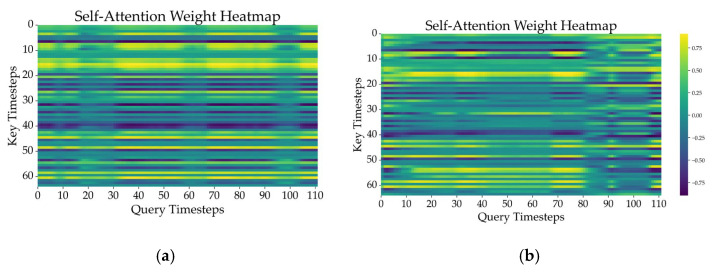
Attention weight heatmaps of representative samples in (**a**) non-fatigue and (**b**) fatigue states.

Figure 9 compares the attention weight heatmaps of representative samples from each class, visually illustrating temporal interaction patterns between fatigue and non-fatigue states. In the non-fatigue state, attention tends to focus on specific timesteps, whereas in the fatigue state, attention is distributed more evenly across the entire sequence. These heatmaps reveal clearly distinguishable attention patterns between the two conditions. By selectively emphasizing critical information dispersed throughout the time series, the model highlights key temporal segments by identifying the time intervals that contribute most significantly to classification.

## 4. Discussion

This study demonstrates the efficacy of a hybrid CNN-LSTM-Attention model in classifying physical fatigue states using multimodal EMG and IMU signals. Comb. 3, utilizing only bilateral GL EMG signals with just two features, achieved a subject-independent accuracy of 0.8794 and a fatigue recall of 0.8476. This result indicates that the model effectively detects fatigue-induced asymmetry in muscle activity, consistent with biomechanical and neuromuscular effects of fatigue reported by Penedo et al. [40], Satas et al. [41], and Heil et al. [42].

Penedo et al. [40] observed that fatigue disrupts the neuromuscular system, reducing force generation capacity and increasing co-contraction asymmetry, as evidenced by an elevated mid-frequency symmetry index in the medial gastrocnemius. This effect likely extends to the adjacent GL muscle. Satas et al. [41] found that knee extensor fatigue does not affect bilateral force control stability but increases anti-phase coordination patterns and synergy indices in asymmetric tasks. These findings support the notion that fatigue impairs symmetric muscle activity and prompts task-specific compensatory strategies, which align with our results. Heil et al. [42] reported that fatigue-induced changes in neuromuscular control increase asymmetry in parameters such as jump height and ground reaction forces, suggesting that inter-limb asymmetry could serve as a potential indicator of muscle fatigue.

Fatigue detection was also effective using signals from a single leg. Comb. 13, which integrates thigh and shank IMU signals with GL EMG from one leg, achieved a subject-independent non-fatigue recall of 0.8603 and a fatigue recall of 0.8794. IMU-derived jerk, encompassing linear and angular rates of change, complements EMG by capturing fatigue-related kinematic alterations, thereby enhancing detection accuracy.

These findings are consistent with Zhang et al. [43], who reported significant increases in lower limb acceleration and jerk following squat exercises, and Zhang et al. [44], who noted motor control degradation and rising jerk values in the pelvis, thigh, and shank during fatigue progression. Additionally, Zhang et al. [45] found that fatigue reduces joint range of motion, knee flexion moments, and ankle power while increasing normalized integrated EMG and root-mean-square (RMS) values. These findings highlight the practical utility of combining IMU and EMG for robust fatigue detection by integrating kinematic, kinetic, and neuromuscular changes.

A subject-wise analysis (Figure 7) revealed individual differences in the contributions of IMU and EMG signals. Some subjects exhibited high fatigue detection performance with EMG signals alone, yet performance declined when relying solely on IMU signals. The opposite was also observed in some cases. This variability suggests that fatigue manifests differently across individuals, with some displaying pronounced neuromuscular changes captured by EMG and others showing more evident kinematic shifts detected by IMU. In this regard, Vargas-Valencia et al. [46] noted that, in the case of IMU, axis alignment is a critical factor in analyzing human gait, which can be interpreted as a contributing factor to the performance differences observed across individuals.

Combining IMU and EMG consistently improved overall performance compared to single-modality approaches. This aligns with findings from Pinto-Bernal et al. [22], who stated that removing EMG from the model results in a performance decrease due to the exclusion of critical features directly tied to muscle fatigue, such as RMS EMG signals. Their study emphasized that while IMU data effectively captures kinematic alterations, the absence of EMG diminishes the ability to detect subtle neuromuscular fatigue indicators, underscoring the complementary value of integrating both modalities for robust fatigue detection across diverse individuals.

The relationship between feature quantity and performance also merits consideration. No direct correlation exists between feature count and subject-independent accuracy. For example, Comb. 14 with 20 features achieved 0.8444, whereas Comb. 3 with 2 features reached 0.8794. This indicates that simply increasing features does not ensure better outcomes. Lazcano et al. [47] observed that excessive data in time-series prediction can lead to overfitting and degraded metrics, while Cronin [48] noted that large volumes of low-quality data impair deep neural network performance, whereas smaller, high-quality datasets yield superior results.

The performance of Comb. 13 (10 features, accuracy 0.8698 and fatigue recall 0.8794) and Comb 3 (2 features, accuracy 0.8794 and fatigue recall 0.8476) reinforces that qualitative feature selection and inter-signal synergy outweigh sheer feature quantity in driving classification success. Considering the potential consequences of overlooking fatigue, minimizing false negatives for the fatigued class should take precedence over slightly improved overall accuracy. From this perspective, the superior recall of Comb. 13 for the fatigued class offers a clear advantage, making it more suitable for applications in occupational safety, rehabilitation, or fatigue monitoring.

As summarized in Table 4, our proposed CNN-LSTM-Attention model demonstrated superior fatigue detection performance, achieving 0.9841 accuracy in 9-fold cross-validation and 0.8794 in LOSOCV.

Given the relatively small dataset, the model risked overfitting to individual-specific characteristics. To mitigate this, we employed strategies such as early stopping, learning rate reduction, and LOSOCV to enhance generalization across subjects. As shown in Figure 8, the training accuracy reached 0.9950, while the validation accuracy stabilized at 0.9517, with a small gap of 0.0433. Additionally, the consistently decreasing training and validation loss curves confirm effective learning without overfitting.

Most existing gait-based fatigue detection studies have been conducted in controlled laboratory environments, such as treadmill walking. Related studies include conventional machine learning models using ECG, IMU with plantar force, or EMG with IMU data [13,21,22]. Among these, the highest-performing study utilized an SVM model with IMU and plantar force sensors, achieving 0.99 accuracy [21]. However, this study was limited to a small sample of 18 participants, focusing on an elderly population (mean age 63.4 ± 4.1), which is relatively more prone to fatigue, thereby restricting the generalizability of its findings.

In contrast, this study not only includes 35 participants from a broader, socially active age group (mean age 32.14 ± 9.73 years, ranging from their 20s to 40s) but collects data in a natural environment, incorporating fatigue-inducing tasks such as over-ground walking and repetitive exercise. Prior studies by Aoki et al. [20] and Baghdadi et al. [49] explored similar tasks. However, our approach demonstrated superior performance, surpassing the results of these prior studies. Also, Aoki et al. [20] used Kinect sensors and included only eight participants, resulting in spatial constraints and limited statistical power.

These results underscore the benefits of natural over-ground gait assessment, multimodal fusion of EMG and IMU data through a CNN-LSTM-Attention architecture, systematic sensor configuration optimization, and rigorous subject-independent validation. These innovations collectively address key limitations of traditional single-modality or subject-specific models.

Since this study was conducted with healthy adults, its applicability to other populations, such as patients or specific groups, remains to be verified. This study focuses solely on physical fatigue without exploring its interactions with mental fatigue. Moreover, the emphasis on short-term induced fatigue leaves long-term cumulative fatigue, such as that accrued over a day, unaddressed. In addition, the computational cost and latency of the proposed hybrid CNN-LSTM-Attention model were not evaluated. Future work should investigate the model’s efficiency and responsiveness, especially in wearable or edge-computing environments, to ensure its practicality for real-world applications.

As shown in Figure 9, the attention mechanisms provide valuable interpretability insights. However, further research is needed to fully validate the explainability of the attention mechanisms.

Nevertheless, these findings hold substantial real-world implications, particularly for wearable-based fatigue monitoring. Implementing fatigue monitoring through wearables like smartwatches could improve workplace health, enable personalized rest interventions in industrial environments, optimize sports training, and support individualized rehabilitation, ultimately reducing accident risks and long-term health issues.

## 5. Conclusions

This study demonstrates the potential of a multimodal fatigue detection system integrating sEMG and IMU signals within a hybrid CNN-LSTM-Attention framework. Notably, Comb. 13 achieves an accuracy of 0.8698 with 10 features and a recall of 0.8794 for the fatigued class, ensuring robust performance across diverse subjects. This performance is supported by stable convergence, with training accuracy reaching 0.9950 and validation accuracy stabilizing at 0.9517, confirming its technical reliability. Unlike traditional subject-dependent approaches, the system exhibits exceptional generalizability, validated through LOSOCV, enabling applications in occupational safety, sports training, and rehabilitation.

These findings highlight that optimal fatigue detection depends on synergistic signal combinations rather than feature quantity, with the hybrid model effectively capturing spatial, temporal, and attention-weighted fatigue indicators.

Future work should explore longitudinal fatigue tracking, integration of additional physiological signals, and testing in naturalistic environments to further enhance scalability and applicability. Incorporating modalities like heart rate monitoring, eye tracking, or respiratory pattern analysis could bridge the dimensions of physical and mental fatigue. Including participants with varied physical conditions, such as those in exercise or rehabilitation programs, would enable comparative analysis and broaden the findings’ applicability. Testing in naturalistic settings rather than controlled laboratory environments supports the development of practical fatigue detection systems.

## Figures and Tables

**Table 1:** Sensor signal combinations and feature configurations.

Category	Combination	Sensor and Signal Type
Bilateral vs. unilateral GL EMG	1	$GL_{L}\;(EMG)$
	2	$GL_{R}\;(EMG)$
	3	$GL_{L}\;and\;GL_{R}\;(EMG)$
TA jerk	4	$TA_{L}\;and\;TA_{R}\;(linear\;jerk)$
	5	$TA_{L}\;and\;TA_{R}\;(angular\;jerk)$
	6	$TA_{L}\;and\;TA_{R}\;(linear\;jerk,\;angular\;jerk)$
RF jerk	7	$RF_{L}\;and\;RF_{R}\;(linear\;jerk)$
	8	$RF_{L}\;and\;RF_{R}\;(angular\;jerk)$
	9	$RF_{L}\;and\;RF_{R}\;(linear\;jerk,\;angular\;jerk)$
Single leg hybrid	10	$GL_{L}\;\left(EMG\right),\;TA_{L}\left(linear\;jerk,\;angular\;jerk\right)$
	11	$GL_{L}\;\left(EMG\right),RF_{L}(linear\;jerk)$
	12	$TA_{L}(linear\;jerk,\;angular\;jerk)$,$\;RF_{L}(linear\;jerk)$
	13	$\;GL_{L}\;\left(EMG\right),\;TA_{L}\;\left(linear\;jerk,\;angular\;jerk\right),\;RF_{L}\;(linear\;jerk)$
Comprehensive	14	$GL_{L}\;and\;GL_{R}\;\left(EMG\right)$, $TA_{L}\;and\;TA_{R}\;(linear\;jerk,\;angular\;jerk)$, $RF_{L}\;and\;RF_{R}\;(linear\;jerk)$

**Table 2:** Subject-independent and subject-dependent accuracy by signal combination.

Category	Comb.	Sensors	Features	LOSOCV Acc.	9-Fold Acc.
Bilateral vs. unilateral GL EMG	1	1	1	0.8095	0.8444
	2	1	1	0.8397	0.8841
	3	2	2	0.8794	0.9127
TA jerk	4	2	6	0.7492	0.9492
	5	2	6	0.7429	0.9524
	6	2	12	0.7619	0.9587
RF jerk	7	2	6	0.7381	0.8508
	8	2	6	0.6984	0.9381
	9	2	12	0.6968	0.9460
Single leg hybrid	10	2	7	0.8587	0.9762
	11	2	4	0.8365	0.9365
	12	2	9	0.7730	0.9571
	13	3	10	0.8698	0.9698
Comprehensive	14	6	20	0.8444	0.9841

**Table 3:** Classification performance metrics for selected sensor combinations under subject-independent conditions (LOSOCV).

Comb.	State	Precision	Recall	F1-Score	Accuracy
3	Non-fatigued	0.8567	0.9111	0.8831	
	Fatigued	0.9051	0.8476	0.8754	0.8794
6	Non-fatigued	0.7653	0.7556	0.7604	
	Fatigued	0.7586	0.7683	0.7634	0.7619
7	Non-fatigued	0.7551	0.7048	0.7291	
	Fatigued	0.7232	0.7714	0.7465	0.7381
13	Non-fatigued	0.8770	0.8603	0.8686	
	Fatigued	0.8629	0.8794	0.8711	0.8698
14	Non-fatigued	0.8511	0.8349	0.8429	
	Fatigued	0.8380	0.8540	0.8459	0.8444

**Table 4:** Comparative summary of gait-based fatigue detection studies.

Study	Tasks	Number of Participants	Mean Age (Years)	Modality	Model	Cross-Validation	Performance Result
Ni et al. [13]	Treadmill exercise	80	29.1 ± 6.5	ECG	LightGBM	10-fold cross validation	Accuracy 85.5%
Pinto-Bernal et al. [22]	Treadmill exercise	24	21.75 ± 1.16	EMG+IMU	RF	5-fold cross validation	Accuracy 96.5%
Zhang et al. [21]	Treadmill exercise	18	63.4 ± 4.1	IMU+Plantar force	SVM	LOSOCV	Accuracy 99%
Baghdadi et al. [49]	Walking and manufacturing task	20	37.1 ± 17.5	IMU	SVM	5-fold cross validation	Accuracy 90%
Aoki et al. [20]	Walking and exercise	8	23.0 ± 0.76	Kinect sensor 2	RNN	LOSOCV	AUC 86.0%
This study	Walking and exercise	35	32.14 ± 9.73	sEMG+IMU	CNN-LSTM-Attention	9-fold cross validation	Accuracy 98.41%
						LOSOCV	Accuracy 87.94%

## Data Availability

The data used in this study are available upon request from the corresponding author. The data are not publicly available due to participant confidentiality.
